# Molecular evolution of Azagny virus, a newfound hantavirus harbored by the West African pygmy shrew (*Crocidura obscurior*) in Côte d'Ivoire

**DOI:** 10.1186/1743-422X-8-373

**Published:** 2011-07-28

**Authors:** Hae Ji Kang, Blaise Kadjo, Sylvain Dubey, François Jacquet, Richard Yanagihara

**Affiliations:** 1 John A. Burns School of Medicine, University of Hawaii at Manoa, Honolulu, HI 96813, USA; 2 Department of Biology, Université de Cocody, Abidjan, Côte d'Ivoire; 3 School of Biological Sciences, University of Sydney, New South Wales 2006, Australia; 4 Department Systématics and Evolution, Muséum National d'Histoire Naturelle, Paris, France

## Abstract

**Background:**

Tanganya virus (TGNV), the only shrew-associated hantavirus reported to date from sub-Saharan Africa, is harbored by the Therese's shrew (*Crocidura theresae*), and is phylogenetically distinct from Thottapalayam virus (TPMV) in the Asian house shrew (*Suncus murinus*) and Imjin virus (MJNV) in the Ussuri white-toothed shrew (*Crocidura lasiura*). The existence of myriad soricid-borne hantaviruses in Eurasia and North America would predict the presence of additional hantaviruses in sub-Saharan Africa, where multiple shrew lineages have evolved and diversified.

**Methods:**

Lung tissues, collected in RNAlater^®^, from 39 Buettikofer's shrews (*Crocidura buettikoferi*), 5 Jouvenet's shrews (*Crocidura jouvenetae*), 9 West African pygmy shrews (*Crocidura obscurior*) and 21 African giant shrews (*Crocidura olivieri*) captured in Côte d'Ivoire during 2009, were systematically examined for hantavirus RNA by RT-PCR.

**Results:**

A genetically distinct hantavirus, designated Azagny virus (AZGV), was detected in the West African pygmy shrew. Phylogenetic analysis of the S, M and L segments, using maximum-likelihood and Bayesian methods, under the GTR+I+Γ model of evolution, showed that AZGV shared a common ancestry with TGNV and was more closely related to hantaviruses harbored by soricine shrews than to TPMV and MJNV. That is, AZGV in the West African pygmy shrew, like TGNV in the Therese's shrew, did not form a monophyletic group with TPMV and MJNV, which were deeply divergent and basal to other rodent- and soricomorph-borne hantaviruses. Ancestral distributions of each hantavirus lineage, reconstructed using Mesquite 2.74, suggested that the common ancestor of all hantaviruses was most likely of Eurasian, not African, origin.

**Conclusions:**

Genome-wide analysis of many more hantaviruses from sub-Saharan Africa are required to better understand how the biogeographic origin and radiation of African shrews might have contributed to, or have resulted from, the evolution of hantaviruses.

## Background

Sub-Saharan Africa has long been considered the "birthplace" of many medically important vector-borne and zoonotic viruses. Among myriad examples are human immunodeficiency virus type 1, which resulted from multiple cross-species transmissions among nonhuman primates to become well established in humans [1,2]; Ebola virus, a filovirus harbored by fruit bats [3,4], which is among the deadliest viruses on earth; and Lassa virus, a rodent-borne arenavirus [5], which causes an estimated 100,000-500,000 human infections each year in West African countries. In addition, the recent report of Lujo virus as the cause of fatal hemorrhagic fever in South Africa [6], where severe arenavirus-associated human disease had not been previously recognized, serves as a startling reminder that other pathogenic zoonotic viruses remain undiscovered.

While rodents (Order Rodentia) have long been known to serve as reservoirs of hantaviruses (Family Bunyaviridae, Genus Hantavirus), recent studies indicate a far richer genetic diversity among hantaviruses harbored by shrews (Order Soricomorpha, Family Soricidae) and moles (Family Talpidae) of multiple species spanning across four continents, including Thottapalayam virus (TPMV) in the Asian house shrew (*Suncus murinus*) [7,8], Imjin virus (MJNV) in the Ussuri white-toothed shrew (*Crocidura lasiura*) [9], Cao Bang virus (CBNV) in the Chinese mole shrew (*Anourosorex squamipes*) [10], Seewis virus (SWSV) in the Eurasian common shrew (*Sorex araneus*) [11,12], Ash River virus (ARRV) in the masked shrew (*Sorex cinereus*) and Jemez Spring virus (JMSV) in the dusky shrew (*Sorex monticolus*) [13], Kenkeme virus (KKMV) in the flat-skulled shrew (*Sorex roboratus*) [14], Camp Ripley virus (RPLV) in the northern short-tailed shrew (*Blarina brevicauda*) [15], Asama virus (ASAV) in the Japanese shrew mole (*Urotrichus talpoides*) [16], Oxbow virus (OXBV) in the American shrew mole (*Neurotrichus gibbsii*) [17], Nova virus (NVAV) in the European common mole (*Talpa europaea*) [18] and Rockport virus (RKPV) in the eastern mole (*Scalopus aquaticus*) [19]. Until recently, conspicuous in their absence have been reports of hantaviruses in Africa. Sangassou virus (SANV) in the African wood mouse (*Hylomyscus simus*) [20] and Tanganya virus (TGNV) in the Therese's shrew (*Crocidura theresae*) [21] are the only known examples to date, but additional hantaviruses likely exist in sub-Saharan Africa, where unique rodent and soricid lineages have evolved and diversified.

In this study, lung tissues from crocidurine shrews captured in Côte d'Ivoire were systematically analyzed for hantavirus RNA by reverse transcription polymerase chain reaction (RT-PCR). Undaunted by the vast genetic diversity of hantaviruses harbored by shrews, we resorted to a brute-force strategy of designing oligonucleotide primers based on regions of high sequence conservation. After exhaustive hit-and-miss attempts, a genetically distinct hantavirus, designated Azagny virus (AZGV), was detected in the West African pygmy shrew (*Crocidura obscurior*). Sequence and phylogenetic analyses of the S, M and L segments indicated that AZGV shared a common ancestry with TGNV and was evolutionarily distant from TPMV and MJNV, two crocidurine shrew-borne hantaviruses in Asia.

## Methods

### Trapping and specimen collection

**Figure 1:**
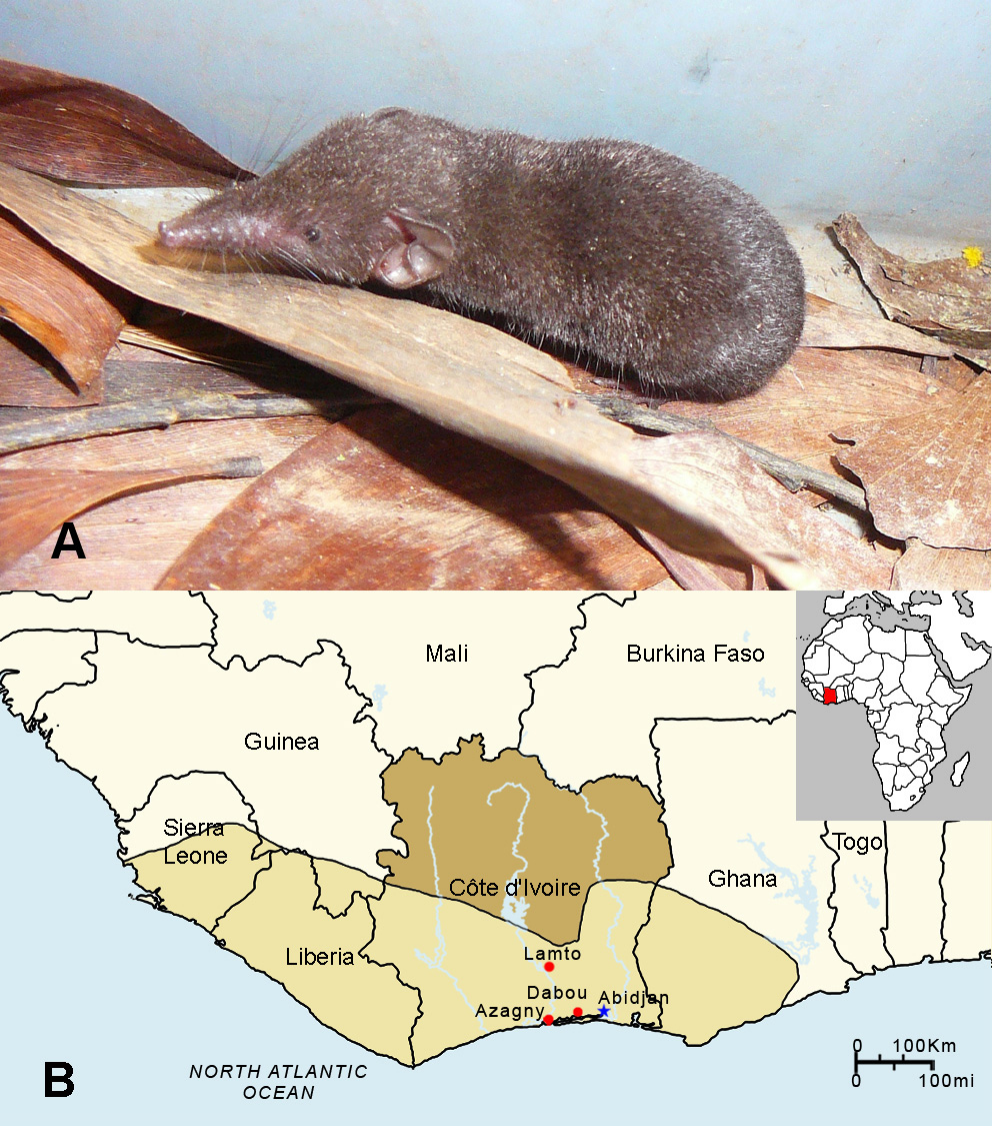
**West African pygmy shrew and geographic distribution**. (A) *Crocidura obscurior *(West African pygmy shrew). (B) Map of Côte d'Ivoire, showing sites where crocidurine shrews were captured in 2009, particularly Azagny National Park, where a West African pygmy shrew harboring a newfound hantavirus was captured. The geographic range of the West African pygmy shrew extends along the coast of Côte d'Ivoire and neighboring Ghana, Guinea, Liberia and Sierra Leone (shaded area).

Shrews were live caught, using pitfall traps, as well as Sherman traps (8 × 9 × 23 cm) (H.B. Sherman, Tallahassee, FL) and Longworth traps (14 × 6.5 × 8.5 cm) (Penlon Ltd., Oxford, UK). Pitfall traplines were set with drift fences using 1-m plastic sheeting or fine mesh cloth erected between short poles and running across plastic buckets that were 20-cm deep and buried flush with the soil surface. Up to 20 buckets were placed along 100- to 150-m long drift fences at 5- to 10-m intervals. Sherman and Longworth traps were set at intervals of 5 m during daylight hours of each day. Trapping was conducted over two to 10 consecutive days, at Azagny National Park, Lamto Natural Reserve and Dabou (Figure 1), during March, July, August, November and December 2009. Traplines and pitfalls were checked every morning and coordinates of each trapped animal were recorded by the global positioning system (GPS). Species, gender, weight, reproductive maturity and GPS coordinates of each captured shew were recorded. Lung tissues were collected in RNAlater^® ^RNA Stabilization Reagent (Qiagen, Inc., Chatsworth, CA) and processed for RNA extraction, within 4 weeks of collection, for RT-PCR analysis. All experimental procedures on animals followed internationally recognized guidelines and were approved by the Ivorian National Park Office and the Muséum National d'Histoire Naturelle.

### RNA extraction and cDNA synthesis

Total RNA was extracted, using the PureLink Micro-to-Midi total RNA purification kit (Invitrogen, San Diego, CA), from shrew tissues, and cDNA was prepared using the SuperScript III First-Strand Synthesis System (Invitrogen) and random hexamers and/or an oligonucleotide primer (OSM55: 5'-TAGTAGTAGACTCC-3') designed from the conserved 5'-end of the S, M and L segments of hantaviruses.

### RT-PCR and DNA sequencing

PCR was performed as described previously [18], with each 20-μL reaction containing 250 μM dNTP, 2 mM MgCl_2_, 1 U of AmpliTaq polymerase (Roche, Basel, Switzerland) and 0.25 μM of oligonucleotide primers, with trial-and-error testing of primers and modified cycling conditions. Initial denaturation at 94°C for 5 min was followed by two cycles each of denaturation at 94°C for 40 sec, two-degree step-down annealing from 48°C to 38°C for 40 sec, and elongation at 72°C for 1 min, then 32 cycles of denaturation at 94°C for 40 sec, annealing at 42°C for 40 sec, and elongation at 72°C for 1 min, in a GeneAmp PCR 9700 thermal cycler (Perkin-Elmer, Waltham, MA). Amplicons were purified using the QIAQuick Gel Extraction Kit (Qiagen, Hilden, Germany), and DNA sequencing was performed using an ABI Prism 377XL Genetic Analyzer (Applied Biosystems, Foster City, CA).

### Protein analysis and secondary structure prediction

For secondary structure prediction of the nucleocapsid protein and envelope glycoprotein, amino acid sequences were submitted to NPS@ structure server [22]. Glycosylation sites and transmembrane helices were predicted using NetNlyc 1.0 and Predictprotein [23] and TMHMM version 2.0 [24], respectively.

### Phylogenetic analysis

Partial S-, M- and L-segment sequences of AZGV were aligned and compared with publicly available hantavirus sequences, using ClustalW (DNASTAR, Inc., Madison, WI) [25] and transAlign [26]. Representative members of the *Nairovirus *(Dugbe virus, NC_004157) and *Phlebovirus *(Rift Valley fever virus, NC_014395) genera were included as outgroups. Phylogenetic trees were generated by maximum likelihood and Bayesian methods, implemented in PAUP* (Phylogenetic Analysis Using Parsimony, 4.0b10) [27], RAxML Blackbox webserver [28] and MrBayes 3.1 [29], under the best-fit GTR+I+Γ model of evolution established using jModeltest 0.1.1 [30]. By employing two complementary and not redundant ML methods, additional analytical rigor was achieved. Topologies were evaluated by bootstrap analysis of 1,000 iterations, and posterior node probabilities were based on 2 million generations and estimated sample sizes over 100 (implemented in MrBayes). Since tree topologies for each genomic segment were very similar using RAxML, PAUP* and MrBayes, trees generated by MrBayes were displayed.

### Biogeographic analyses

Ancestral distributions of each lineage were reconstructed using a maximum-likelihood approach using Mesquite 2.74 [31]. The current geographic distribution of hantavirus species was coded as 0 for Eurasia, 1 for Africa, and 2 for America. Similarly, we tested for an ancestral Soricomorpha (0) vs. Rodentia (1) origin of hantaviruses. The model of character evolution was a simple stochastic model (Mk1) [32], which assumed an equal and symmetrical rate of change between any two states [33,34], and the character state frequencies were estimated from the transition probabilities. In this likelihood-based approach, the probability that a character changes along a branch of the tree is consequently a function of the branch length, with changes being less likely along shorter branches than longer ones.

To have branch lengths representing absolute time of divergence, a calibrated Bayesian tree was built with BEAST 1.4 [35] using a Yule tree prior (adequate to study interspecific diversification). Preliminary analyses were performed with an uncorrelated lognormal relaxed clock to test if a strict molecular clock can be rejected for our dataset (ucld.stdev parameter with a frequency histogram abutting 0). Because in our simulations the ''ucld.stdev'' had a frequency histogram not abutting 0, we chose this molecular clock for the analyses [36]. The analyses were performed with two independent chains and 10 million generations and chains were sampled every 1,000 generations with a burn-in of 1 million generations. Convergences were determined with Tracer v1.4 [37].

### mtDNA sequence analysis

The entire 1,140-nucleotide region of the cytochrome *b *gene of mtDNA was amplified by PCR, as previously described [17,18], to confirm the taxonomic identity of the hantavirus-infected shrew and to examine its phylogenetic relationship to other shrew hosts.

## Results and Discussion

### RT-PCR and sequence analysis

In analyzing lung tissues from 39 Buettikofer's shrews (*Crocidura buettikoferi*), 5 Jouvenet's shrews (*Crocidura jouvenetae*), 9 West African pygmy shrews (*Crocidura obscurior*) and 21 African giant shrews (*Crocidura olivieri*) by RT-PCR using multiple primer pairs, novel hantavirus RNAs were detected in a West African pygmy shrew, captured in Azagny National Park (latitude, 5°14.5' N; longitude, 4°48.1' W), Côte d'Ivoire, on December 15, 2009 (Figure 1). The identity of the West African pygmy shrew (GenBank JF276229), in which AZGV was detected, was confirmed by phylogenetic analysis of the 1,140-nucleotide mtDNA cytochrome *b *gene (results not shown).

Although hantavirus RNAs were not found in tissues of the other three African crocidurine shrew species tested, we believe it is simply because suitable oligonucleotide primers were not designed. This conjecture is based on our past experience of repeated failed attempts before the successful detection of soricomorph-borne hantaviruses [17-19]. Thus, continuing efforts are ongoing to retest these specimens, and future expeditions are being planned to collect tissues suitable for virus isolation attempts.

Despite intensive efforts to obtain the full genome of AZGV, the inability to effectively design primers for this novel hantavirus and the limited amount of available RNA were obstacles too great to overcome. Nevertheless, pairwise alignment and comparison of partial S-, M- and L-genomic segment sequences of AZGV with representative rodent- and soricid-borne hantaviruses clearly demonstrated that AZGV was genetically distinct (Table 1). AZGV was most closely related to TGNV from neighboring Guinea and was highly divergent from TPMV and MJNV, two crocidurine shrew-borne hantaviruses from Asia.

**Table 1:** Sequence similarities (%) of the partial S, M and L segments of AZGV strain KBM15 and representative hantaviruses harbored by rodents and soricomorphs

	S segment		M segment		L segment	
			
Virus strain	540 nt	180 aa	687 nt	229 aa	4548 nt	1516 aa
HTNV 76-118	60.2	59.4	65.4	68.2	69.1	73.3
SOOV SOO-1	59.2	60.0	65.3	67.4	68.4	73.4
DOBV Greece	62.7	60.6	67.8	66.9	67.7	71.5
SEOV 80-39	61.9	60.0	64.6	67.8	68.2	71.9
PUUV Sotkamo	62.4	63.3	58.1	50.0	66.2	67.9
TULV 5302v	62.0	63.9	58.3	50.0	65.4	67.1
PHV PH-1	64.4	64.4	54.7	46.3	64.7	67.3
SNV NMH10	59.3	58.1	57.7	52.9	65.7	67.0
ANDV Chile9717869	61.4	59.2	56.9	52.9	65.4	66.5
CBNV CBN-3	64.2	63.3	68.5	73.1	71.8	77.8
ARRV MSB73418	60.7	62.7	-	-	70.4	76.1
JMSV MSB144475	63.3	66.1	67.0	71.9	70.8	77.8
SWSV mp70	59.8	61.7	66.7	69.4	69.3	74.7
KKMV MSB148794	61.9	62.2	68.1	71.2	71.0	77.7
RPLV MSB89863	51.8	44.2	64.8	65.7	69.9	74.4
TGNV Tan826	63.5	62.1	-	-	78.7	88.3
MJNV Cl05-11	53.0	47.2	54.1	45.2	61.3	61.1
TPMV VRC66412	55.1	45.5	52.3	41.5	60.9	60.8
OXBV Ng1453	63.6	64.8	64.0	62.0	70.1	74.8
ASAV N10	62.4	63.9	64.1	65.7	71.0	76.2
NVAV MSB95703	58.1	50.3	51.8	41.0	63.6	61.3

Abbreviations: ANDV, Andes virus; ARRV, Ash River virus; ASAV, Asama virus; CBNV, Cao Bang virus; DOBV, Dobrava virus; HTNV, Hantaan virus; JMSV, Jemez Spring virus; KKMV, Kenkeme virus; MJNV, Imjin virus; NVAV, Nova virus; OXBV, Oxbow virus; PHV, Prospect Hill virus; PUUV, Puumala virus; RPLV, Camp Ripley virus; SEOV, Seoul virus; SNV, Sin Nombre virus; SOOV, Soochong virus; SWSV, Seewis virus; TGNV, Tanganya virus; TPMV, Thottapalayam virus; TULV, Tula virus. nt, nucleotides; aa, amino acids.

In a 540-nucleotide region of the AZGV S segment (GenBank JF276226), the hypothetical nonstructural protein (NSs) open-reading frame, found in arvicolid, neotomine and sigmodontine rodent-borne hantaviruses, was absent. Analysis of a 687-nucleotide region of the AZGV M-genomic segment (GenBank JF276227), which spanned the Gn-Gc juncture, showed the highly conserved WAASA amino acid motif cleavage site at positions 167 to 171. Potential N-linked glycosylation sites were found at amino acid positions 46, 118 and 142 in the Gn glycoprotein and 179 and 216 in the Gc gylcoprotein. Analysis of the nearly full-length 4,548-nucleotide AZGV L-genomic segment (GenBank JF276228), encoding an incomplete RNA-dependent RNA polymerase of 1,516 amino acids, showed conservation of the five major motifs, designated A, B, C, D and E [38].

Extensive analysis of the available AZGV genome, using multiple recombination-detection methods, including GENECONV, Bootscan, Chimaera, 3SEQ, RDP, SiScan, MaxChi and HyPhy Single Recombinant Breakpoint [39], failed to disclose any evidence of genetic recombination.

### Phylogenetic analysis and co-phylogeny mapping

**Figure 2:**
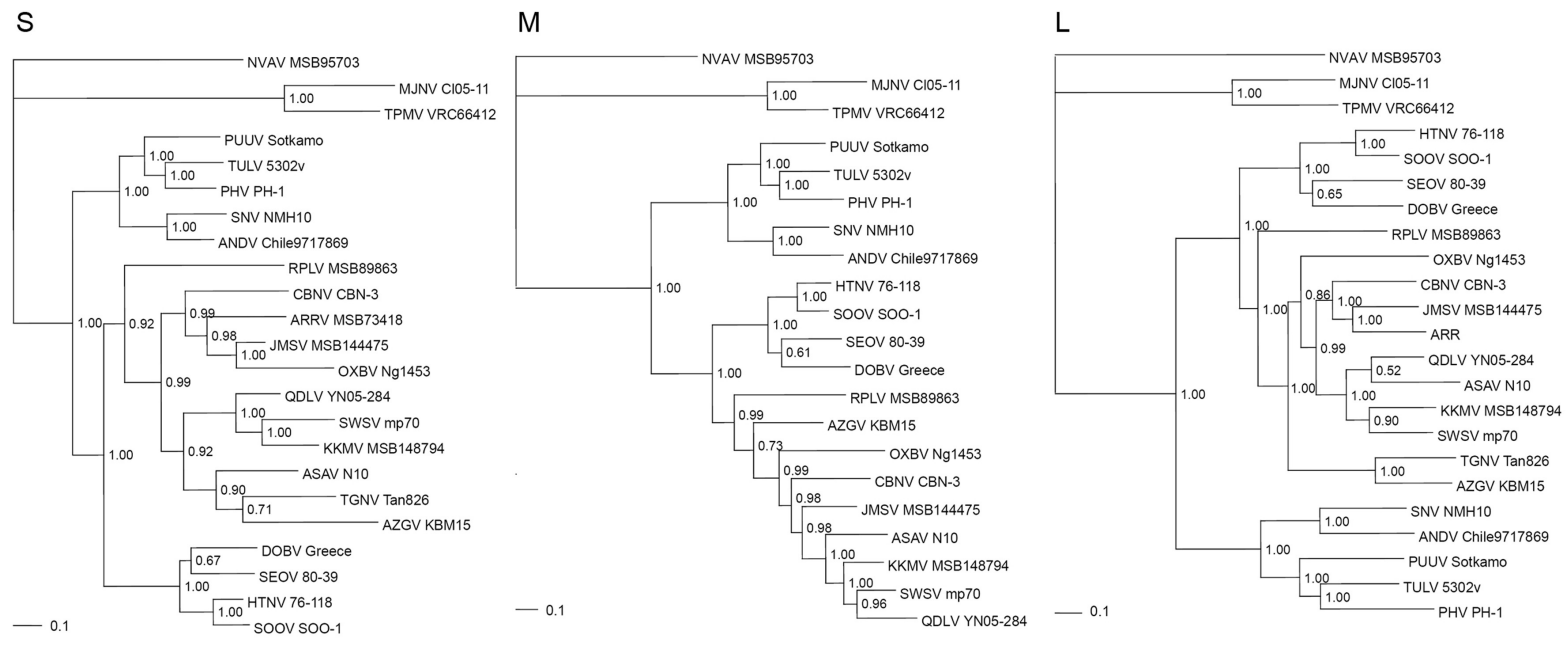
**Phylogenetic trees**. Similar tree topologies, based on the coding regions of the partial (S) 540-nucleotide S-, (M) 687-nucleotide M- and (L) 4,548-nucleotide L-genomic segments of AZGV, were generated by maximum-likelihood and Bayesian methods, under the GTR+I+Γ model of evolution. In these unrooted trees, the phylogenetic positions of AZGV are shown in relation to soricomorph-borne hantaviruses, including Tanganya virus (TGNV Tan826: EF050455, EF050454), Imjin virus (MJNV Cl05-11: EF641804, EF641798, EF641806), Thottapalayam virus (TPMV VRC66412: AY526097, EU001329, EU001330), Cao Bang virus (CBNV CBN-3: EF543524, EF543526, EF543525), Ash River virus (ARRV MSB73418: EF650086, EF619961), Jemez Springs virus (JMSV MSB144475: FJ593499, FJ593500, FJ593501), Seewis virus (SWSV mp70: EF636024, EF636025, EF636026), Kenkeme virus (KKMV MSB148794: GQ306148, GQ306149, GQ306150), Qiandao Lake virus (QDLV YN05-284: GU566023, GU566022, GU566021), Camp Ripley virus (RPLV MSB89863: FJ790772, EF540774, EF540771), Asama virus (ASAV N10: EU929072, EU929075, EU929078), Oxbow virus (OXBV Ng1453: FJ539166, FJ539167, FJ593497) and Nova virus (NVAV MSB95703: FJ539168, HQ840957, FJ593498). Also shown are Hantaan virus (HTNV 76-118: NC_005218, Y00386, NC_005222), Soochong virus (SOOV SOO-1: AY675349, AY675353, DQ562292), Dobrava virus (DOBV Greece: NC_005233, NC_005234, NC_005235), Seoul virus (SEOV HR80-39: NC_005236, NC_005237, NC_005238), Tula virus (TULV M5302v: NC_005227, NC_005228, NC_005226), Puumala virus (PUUV Sotkamo: NC_005224, NC_005223, NC_005225), Prospect Hill virus (PHV PH-1: Z49098, X55129, EF646763), Andes virus (ANDV Chile-9717869: NC_003466, NC_003467, NC_003468) and Sin Nombre virus (SNV NMH10: NC_005216, NC_005215, NC_005217). The numbers at each node are posterior probabilities, and the scale bars indicate nucleotide substitutions per site.

High bootstrap support for a shared ancestry between AZGV and TGNV, as evidenced by bootstrap values of >70% and posterior node probabilities of >0.70, was found in phylogenetic trees, based on available S- and L-segment sequences, using the maximum-likelihood (ML) and Bayesian methods (Figure 2). That is, AZGV in the West African pygmy shrew, like TGNV in the Therese's shrew [21], did not form a monophyletic group with TPMV in the Asian house shrew [8] and MJNV in the Ussuri white-toothed shrew [9], which were deeply divergent and basal to other rodent- and soricomorph-borne hantaviruses, with longer branch lengths suggesting greater evolutionary change and possibly older age.

Phylogenetic trees, reconstructed for co-phylogeny mapping using TreeMap 2.0β [40], exhibited high bootstrap support for each genomic segment (at both the nucleotide and amino acid levels), with segregation of hantaviruses, according to the Subfamily of their soricomorph reservoir hosts. That is, congruent topologies were found in the host-virus phylogenetic relationships, except for previously reported hantaviruses hosted by two species of shrew moles [16,17].

### Biogeographic analyses

**Figure 3:**
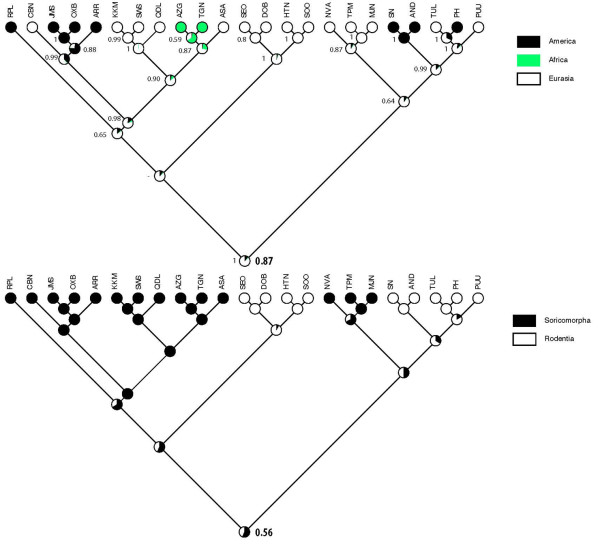
**Phylogenetic distribution and reconstruction**. Phylogenetic distribution and reconstruction of ancestral states for (A) the biogeographic origin (America, Eurasia or Africa) and (B) the host (Soricomorpha or Rodentia) of hantaviruses using Mesquite 2.74 (with a representation of ML supports for each state and node, and ML support [in bold] for the basal nodes). Bayesian posterior probabilities from BEAST are shown for each node in (A).

Because the choice of basal taxa can strongly influence the biogeographic reconstruction of the target taxa in such analyses, the outgroups (Dugbe virus and Rift Valley fever virus) were not included in the analyses. As shown in Figure 3, the common ancestor of all hantaviruses likely emerged in Eurasia rather than Africa (ML support, 87%). Moreover, the analyses suggested that the ancestral hosts of hantaviruses may have been members of the Soricomorpha, rather than Rodentia (ML support, 56%). However, genomic analyses of many more soricomorph-borne hantaviruses, and studies in search of hantaviruses in other potential reservoir hosts, especially insectivorous bats (Order Chiroptera), are necessary to validate these tentative conclusions.

Biogeographic reconstruction, based on fossil records and assuming equally probable exchanges between continents in both directions, suggests that the Family Soricidae originated in Eurasia [33]. And based on karyologic, paleontologic and molecular genetic data, a Palaearctic-Oriental origin has been proposed for the genus *Crocidura*, dating to the Upper Miocene (6.8 million years before present) [34]. Moreover, in-depth analysis of 3,560-base pairs of mitochondrial and nuclear DNA of crocidurine shrews from across Eurasia and Africa, reveals that shrews belonging to the genus *Crocidura *segregate into an Afrotropical clade, an Asian clade and an Old World clade, which includes Afrotropical, East Palaearctic-Oriental and West Palaearctic species [34]. *Crocidura obscurior *groups into the Old World clade, while *Crocidura theresae*, as well as *Crocidura buettikoferi, Crocidura jouvenetae *and *Crocidura olivieri*, group in the Afrotropical clade, and *Crocidura lasiura *in the Asian clade.

Apart from Côte d'Ivoire, the West African pygmy shrew is found in subtropical or tropical moist lowland forests in Ghana, Guinea, Liberia, Sierra Leone and possibly Nigeria. A similar West African distribution is noted for *Crocidura buettikoferi *and *Crocidura jouvenetae*, while *Crocidura olivieri *is far more widespread, from west to east across sub-Saharan Africa [41]. Although previous ecological studies had indicated that *Crocidura theresae*, the presumed reservoir of TGNV, is widespread and abundant in Côte d'Ivoire [42,43], this crocidurine shrew species was not captured during any of our multiple trapping expeditions. As such, we were unable to confirm the presence of TGNV in the Therese's shrew or to analyze the genetic diversity and phylogeography of TGNV.

In this regard, future studies are warranted to confirm that the West African pygmy shrew is the true reservoir host of AZGV. Also, whole genome sequence analysis of many more hantaviruses from sub-Saharan Africa are required to gain a better understanding about how the biogeographic origin and radiation of African shrews might have contributed to, or have resulted from, the evolution and cross-species transmission of hantaviruses. Intensified efforts are underway in search of hantaviruses in other shrew species, including *Myosorex *and *Sylvisorex *shrews, which are not found elsewhere and are unique to Africa. Meanwhile, the disproportionately high number of hantaviruses hitherto discovered in soricomorphs over a relatively brief period of searching, and the far greater genetic diversity of shrew- and mole-borne hantaviruses, compared to those harbored by rodents, provide additional support for the emerging concept that soricomorphs may have served as the early reservoirs of ancestral hantaviruses.

## Competing interests

The authors declare that they have no competing interests.

## Authors' contributions

HJK performed the RNA extraction, RT-PCR, and genetic and phylogenetic analyses. BK and FJ conducted the field expeditions and provided the background data on wild-caught shrews. SD conducted the biogeographic analysis and data interpretation. RY conceived the research design, arranged the collaboration and provided scientific oversight. All authors read and approved the final manuscript.
